# Episodic and Semantic Memory Contribute to Familiar and Novel Episodic Future Thinking

**DOI:** 10.3389/fpsyg.2016.01746

**Published:** 2016-11-10

**Authors:** Tong Wang, Tong Yue, Xi Ting Huang

**Affiliations:** Faculty of Psychology, Southwest UniversityChongqing, China

**Keywords:** episodic future thinking, episodic memory, semantic memory, event familiarity, moderating effect

## Abstract

Increasing evidence indicates that episodic future thinking (EFT) relies on both episodic and semantic memory; however, event familiarity may importantly affect the extent to which episodic and semantic memory contribute to EFT. To test this possibility, two behavioral experiments were conducted. In Experiment 1, we directly compared the proportion of episodic and semantic memory used in an EFT task. The results indicated that more episodic memory was used when imagining familiar future events compared with novel future events. Conversely, significantly more semantic memory was used when imagining novel events compared with familiar events. Experiment 2 aimed to verify the results of Experiment 1. In Experiment 2, we found that familiarity moderated the effect of priming the episodic memory system on EFT; particularly, it increased the time required to construct a standard familiar episodic future event, but did not significantly affect novel episodic event reaction time. Collectively, these findings support the hypothesis that event familiarity importantly moderates episodic and semantic memory's contribution to EFT.

## Introduction

People generate future-oriented thoughts frequently in daily life (Szpunar and Tulving, 2010); these thoughts may be abstract or specific (D'Argembeau et al., 2011), and personal or non-personal (Klein, 2013a,b). People may thus think about abstract and non-personal future events (e.g., considering future environmental issues), specific but non-personal events (e.g., an upcoming public event), abstract but personal events (e.g., the occupation one will take up after graduation), and—characteristically—specific and personal future events (e.g., a date with an acquaintance next Saturday). This latter form of imagining is called episodic future thinking (EFT); it involves projecting oneself forwards in time to pre-experience an event that might happen in one's personal future (Atance and O'Neill, 2001; Suddendorf, 2010; Szpunar, 2010). EFT has received considerable attention in the last decade, particularly regarding its cognitive processes (e.g., Hassabis and Maguire, 2007; Schacter and Addis, 2007; Irish and Piguet, 2013; D'Argembeau, 2015).

Two central hypotheses have been put forward to explain EFT's cognitive process. The constructive episodic simulation hypothesis, introduced by Schacter and Addis (2007), proposes that episodic memory (EM) provides memories of previous experiences that we can flexibly extract and recombine into a novel episodic future event. This hypothesis emphasizes the close link between EM and EFT. However, if one imagines EFT as similar to building a house, episodic details extracted from memories may represent the bricks, but this is insufficient: the bricks cannot be assembled into a house without an appropriate schema (Irish and Piguet, 2013). Irish and Piguet (2013) therefore proposed the semantic scaffolding hypothesis, which proposes that semantic memory (SM) importantly facilitates EFT by providing a scaffolding or framework that allows memory retrieval and future thinking. This latter hypothesis therefore implies that successful EFT involves indispensable semantic information, as well as elements of various episodic memories.

Mounting evidence from preliminary research supports episodic memory's critical role in EFT and the constructive episodic simulation hypothesis. For example, clinical studies have found that patients suffering episodic memory impairment also experienced difficulty imagining personal future events (Addis et al., 2009; Lind and Bowler, 2010; Matthew and Lori-Anne, 2011; de Vito et al., 2012a; Brown et al., 2013). Additionally, extensive neuroimaging research suggests that a common brain network including the medial temporal lobe, prefrontal cortex, and posterior parietal cortex underlies both episodic memory and EFT (Okuda et al., 2003; Addis et al., 2007; Schacter et al., 2007, 2008, 2012; Szpunar et al., 2007). Nonetheless, recent findings indicate that SM also significantly contributes to EFT, supporting the semantic scaffolding hypothesis. Studies examining patients with semantic dementia directly support this hypothesis. Semantic dementia is a progressive neurodegenerative condition characterized by the profound and amodal loss of semantic memory, but relatively intact episodic memory. Individuals with semantic dementia experience difficulty imagining future episodic events, implying significant EFT impairment (Irish et al., 2012; Irish and Piguet, 2013). Additionally, functional neuroimaging studies support SM's important role in EFT: some brain regions involved in semantic processing tasks (e.g., the inferior parietal, lateral temporal, medial prefrontal, and posterior cingulate cortices, particularly in the left hemisphere) are also recruited in imagining future events (Binder et al., 2009). Further, behavioral studies examining healthy people suggest that EM and SM jointly contribute to EFT. These studies found that EFT relies on multiple representational systems, with personal semantic knowledge providing a framework before specific episodic details from remembered past events are accessed, thus providing a context that organizes episodic details into coherent themes and sequences (D'Argembeau and Mathy, 2011; D'Argembeau and Demblon, 2012; Demblon and D'Argembeau, 2014). These findings collectively suggest that both EM and SM play important roles in EFT.

It remains unclear if EM or SM is more important to EFT; few studies have directly addressed this question. We hypothesized that event familiarity importantly moderates EM and SM's relative contribution to EFT. We proposed this hypothesis in response to several lines of research. First, in some studies, imagining a familiar event was rated as more clearly represented and containing more sensorial detail than imagining a novel event (Szpunar and McDermott, 2008; Arnold et al., 2011; de Vito et al., 2012b). We speculated that the phenomenological difference between novel and familiar EFT may stem from that imagining familiar events—relative to novel events—depends more on EM, as this might permit the inclusion of greater detail in representation of future events. Second, Irish et al. (2012) found that patients with semantic dementia showed a stronger tendency to represent events that had been previously experienced in their entirety than patients with Alzheimer's disease when imagining their future, despite explicit task instructions requiring them to generate novel events not previously experienced. This may be because the absence of SM particularly inhibits EFT regarding novel future events. Third, a fMRI study found that regions associated with general semantic processing (i.e., the left fusiform gyrus) showed preferential activity as participants imagined novel events relative to familiar events; this may also reflect the nature of familiarity with particular events (Szpunar et al., 2009). In this context, we hypothesized that familiarity would moderate EFT's relative dependence on EM and SM. Specifically, we hypothesized that imagining a familiar event would involve more EM than imagining a novel event, whereas imagining a novel EFT would involve more SM than a familiar EFT. In order to test this hypothesis, two behavioral experiments were conducted. In experiment 1, we directly compared the proportion of EM and SM used in novel and familiar EFT. Experiment 2 was designed to test the results of Experiment 1 by examining if activating EM systems would differentially affect familiar and novel EFT.

## Experiment 1

### Materials and method

#### Participants and design

Participants were 45 undergraduate students (24 females; age range: 19–25 years). A within-participants design was used: participants completed both of two conditions (familiar vs. novel EFT). No participants had previously participated in any similar experiments; participants received a small payment in compensation after completing the experiment. The study protocol was approved by the Southwest University Research Ethics Committee. All participants provided a written indication of informed consent before participating.

#### Materials

A pilot study was conducted to identify suitable familiar and novel settings. Sixty cue words (including nouns and verbs, half familiar and half novel) were selected from the Modern Chinese Dictionary (Lv and Ding, 2012) and the Chinese Affective Words System (Wang et al., 2008). Additionally, all words were constructed as familiar or novel future events (e.g., “graduation” was constructed as “imagining your own graduation day”). These events were presented to 32 college students who would not participate the main experiment; these participants rated their familiarity and emotional valence using a 7-point scale (1 = *not familiar at all*, 7 = *very familiar*; 1 = *very negative*, 7 = *very positive*). Finally, two familiar events (“experiencing an earthquake,” “your own graduation day”) and two novel events (“experiencing a fire,” “climbing Mount Everest”) were selected as event cues. The difference in emotional valence scores between the selected familiar and novel events was insignificant [familiar: *M* = 4.36, *SD* = 0.95; novel: *M* = 4.31, *SD* = 1.03; *t*_(31)_ = 0.39, *p* = 0.70], but their familiarity scores differed significantly [familiar: *M* = 4.10, *SD* = 0.91; novel: *M* = 2.51, *SD* = 1.09; *t*_(31)_ = 7.82, *p* <0.001]. Familiarity scores collected after completing the EFT task supported this result [familiar: *M* = 4.95, *SD* = 1.24; novel: *M* = 3.67, *SD* = 1.63; *t*_(41)_ = 6.75, *p* <0.001].

#### Procedure

The process included three sessions. Session 1 was the EFT task. Following previous research (Szpunar and McDermott, 2008; de Vito et al., 2012b), participants were presented with four sheets of paper, each displaying the instruction to imagine a specific episodic event occurring in their future corresponding to the provided event cue. Participants spent 5 min imagining each event in as much detail as possible and recording what they imagined in writing. Participants then ceased writing and proceeded to Session 2. Session 2 was a memory task adapted from Anderson (2012). During this task, participants segmented their imagined contents into separate single units (each complete sentence represented a single unit). Participants were instructed that if a given unit reminded them of anything in the past when they were thinking about it, they should briefly record the memory details they had consciously used when generating the future details. In Session 3, participants rated the characteristics of the imagined future event using a 7-point scale, following previous research (e.g., Szpunar and McDermott, 2008; de Vito et al., 2012b). Specifically, each event was rated on a sensory-details index (three measures were summed: visual detail, sound detail, smell/taste detail; 1 = none, 7 = a lot), a clarity-of-context index (three measures were summed: clarity of location, clarity of spatial arrangement of objects, clarity of spatial arrangement of people; 1 = vague, 7 = clear), and an index of the subjective experience associated with the mental image (intensity of experience, 1 = none, 7 = high). Each of the four event cues were presented to participants in a Latin Square order.

#### Data preprocessing

Data from three participants were discarded because those participants gave ≥50% invalid responses (e.g., remembering past events rather than imagining future events; failure to adhere to the time limit). One invalid familiar and two invalid novel events were excluded from among the remaining 42 participants' responses. Thus, the proportion of valid data regarding familiar and novel events was 92.2% and 91.1%, respectively. Memories used in imagining future events were coded as EM or SM following Renoult et al. (2012). EM memories characteristically included episodic events, previous single episodes, or repeated or extended events; SM memories characteristically included organized knowledge, autobiographical facts, or abstract self-related knowledge. All memories consciously used in EFT were placed in a common pool and scored at random. The primary scorer was blind to the study's purposes, had undergone extensive training, and had participated in the development of the encoding principle. Following Levine et al. (2002), inter-rater reliability was assessed by randomly selecting 25% of the memories and scoring them using two additional trained scorers who did not otherwise participate in this study. Inter-rater consistency reliability was 0.70–0.85.

### Results

First, differences in phenomenal character between familiar and novel future events were analyzed using paired *T*-tests; this analysis was intended to test the results of previous studies. The results indicated that familiar EFT did not differ significantly from novel EFT regarding the number of sensorial details and the richness of the subjective experience, whereas familiar EFT were rated as more clearly represented than novel EFT (Table 1). We used Bayes factor analysis to test if the non-significant result indicated insufficient power or supported the null hypothesis (Dienes, 2011, 2014). Values of Bayes' factor (*B*) <1/3 support the null hypothesis; values >3 support an alternative hypothesis; other values indicate that the data is unable to differentiate between support of the null hypothesis and insufficient power. Regarding the number of sensorial details, the mean and SE of the difference were −0.12 and 0.101, respectively. Following Dienes (2014), we calculated *B* using a uniform distribution, as this permitted prediction of the maximum effect (uniform from 0 to 6) rather than specifying a plausible predicted effect size P. This yielded *B* = 0.01. Similarly, the *B-*value of subjective experience was 0.22. These results supported the null hypothesis; i.e., that no difference existed between the familiar and novel events regarding sensorial detail or richness of subjective experience.

**Table 1:** **Characteristics of familiar versus novel event representations**.

	**Familiar event**		**Novel event**		**Main effect**	
	***M***	***SD***	***M***	***SD***	***F***	***η^2^***
Proportion of EM	0.75	0.16	0.56	0.17	79.69^***^	0.66
Proportion of SM	0.25	0.16	0.44	0.16	80.62^***^	0.66
Number of sensorial details	4.25	1.14	4.37	1.04	1.43	0.04
Clarity of context	5.25	0.97	4.38	1.01	25.28^**^	0.39
Subjective experience	5.00	1.11	4.73	1.39	2.34	0.06

** *p <0.01*,

*** *p <0.001*.

We speculated that the phenomenological difference between novel and familiar EFT stems from that imagining familiar events—relative to novel events—depends more on EM, as this might permit the more vivid and clear representation of future events. Therefore, we tested if the proportion of EM and SM used in EFT varied depending on familiarity. We found that more EM was used when imagining familiar future events compared with novel future events (Table 1). Conversely, more SM was used in novel EFT relative to familiar EFT. This result supported our hypothesis, indicating familiar EFT involves more EM than novel EFT, whereas novel EFT involves more SM than familiar EFT. Additionally, we found that participants used more EM than SM in both familiar EFTs [*t*_(41)_ = 10.37, *p* <0.001] and novel EFTs [*t*_(41)_ = 2.41, *p* <0.05], implying the overall importance of EM to EFT.

## Experiment 2

Experiment 2 was designed to test if the results of Experiment 1 would remain stable following priming the EM system. Prior priming of a system may facilitate performance in tasks requiring retrieval of information from the same system (Neely and Durgunoǧ, 1985); therefore, based on the results of Experiment 1, we hypothesized that priming the EM system would preferentially facilitate familiar EFT over novel EFT, given that the former relies more heavily on EM. Further, we hypothesized that the facilitation effect would emerge as reducing the time taken to imagine familiar events or enriching the content of familiar events. Finally, the results of Experiment 1 indicated that both familiar EFT and novel EFT rely less on SM than on EM; therefore, we did not include a SM priming condition in this experiment.

### Materials and method

#### Participants and design

This experiment used a 2 (priming condition: control vs. EM priming) × 2 (event familiarity: familiar vs. novel) mixed design. Priming condition was a between-participants factor; event familiarity was a within-participants factor. Participants were 104 undergraduate students (52 female; age range: 19–25 years). All participants provided a written indication of informed consent prior to participating and received a small compensatory payment. No participants had previously participated in any similar experiments. Participants were randomly assigned to conditions using Excel. The independent variables were reaction time and the imagined events' phenomenal characteristics.

#### Materials

##### EFT task

Similarly to Experiment 1, 130 cue words (including nouns and verbs, half familiar and half novel) were selected from the Modern Chinese Dictionary (Lv and Ding, 2012) and the Chinese Affective Words System (Wang et al., 2008) and constructed as future events. In order to choose appropriate EFT cues, 40 participants rated the selected words' familiarity and emotional valence. Five familiar and five novel EFT cue words were subsequently selected. The familiar event cues were “communicating with a foreigner,” “listening to a speech,” “Playing football,” “attending a meeting,” and “taking a cable car.” The novel event cues were “watching an opera,” “one day in the desert,” “going skydiving,” “watching a bullfight,” and “attending a religious service.” Familiar and novel cues differed significantly regarding familiarity [familiar: *M* = 3.00, *SD* = 1.40, novel: *M* = 1.55, *SD* = 0.91, *t*_(39)_ = 6.33, *p* <0.001]^1^, but not regarding emotional valence [familiar: *M* = 3.62, *SD* = 0.98, novel: *M* = 3.50, *SD* = 1.34, *t*_(39)_ = 0.47, *p* = 0.64].

##### Materials for EM priming

The process for EM priming was modified from previous studies and has been supported as priming only the EM system (Philippot et al., 2003; Madore and Schacter, 2014; Jing et al., 2016). Participants recalled a specific important episodic event and then answered several questions inducing them to recall specific details about the people, setting, and actions involved in that event; participants were encouraged to elaborate further on any details mentioned. A voice recorder was used to record the constructed events and elaborations.

#### Procedure

The main experiment was comprised of the memory priming and EFT tasks. First, participants assigned to the EM priming condition completed the provided questionnaire, priming their EM system. Participants began the EFT task immediately afterward. Participants in the control condition performed only the EFT task.

The EFT task procedure was presented on the screen of a Lenovo desktop computer controlled by Eprime2.0 software. The cue-word paradigm (Addis et al., 2007) was adapted for use in this experiment; it proceeded through a construction phase, an elaboration phase, and a rating phase. In the construction phase, participants should construct a standard future event corresponding to the provided cue-word once a red fixation cross appeared (standard future events were specific events that lasted for between several minutes and 24 h and simultaneously contained the following elements: person, location, time, and event). This phase was limited to 40 s, following the pilot study (participants would grow impatient if this phase lasted longer and hurry if it were shorter). Once a standard event was constructed and spoken out, participants should press the “Enter” button immediately, thereby entering the elaboration phase. Particularly, the duration between the appearance of a red fixation cross and the participant's pressing of the “Enter” button was termed the “reaction time” and measured as a dependent variable, similar to Addis et al. (2007, 2011). In the elaboration phase, participants continued to imagine and elaborate on the details of the constructed event for a further 40 s in as much details as possible. A bell would ring to indicate that the time limit had expired; participants would then rate the EFT's phenomenal characteristics using the same questionnaire as in Experiment 1.

It is important to note that EFT task was explained to participants prior to completing the memory-priming task and participants completed some practice trials. The experimenter would examine the results of participants' practice trials regarding the following aspects: if the constructed event was a standard event, if the “Enter” button was pressed immediately once an event had been constructed, and if the elaboration phase was completed. Participants who met each point in their practice trial began the main experiment; participants who did not continued to practice until their practice trial met all three points.

#### Data preprocessing

After excluding invalid data (e.g., misremembered event cues, such as mistaking “watching an opera” for “watching a movie”) and invalid participants (e.g., participants who misremembered ≥50% of event cues), 88 valid subjects' ratings of phenomenal characteristics were retained (46 in the control condition, 25 female; 42 in the EM priming condition, 22 female). The valid response rate was 84.6%. Regarding reaction time data, invalid data (e.g., imagining a non-standard event, misremembering the event cue, failing to press the “Enter” button immediately once a standard event had been constructed) and invalid participants (i.e., participants who gave ≥50% invalid responses) were excluded, leaving 72 valid participants (36 subjects in each condition, 19 and 18 female in the control condition and EM priming condition, respectively). The valid response rate was 69.2%. Interviews conducted after the experiment suggested the task's novelty and difficulty were responsible for the relatively low valid response rate. Participants indicated the instructions' complexity made it quite difficult to adhere to them in every single trial. Additionally, the standards for eliminating invalid data were strict and contributed to reducing the valid response rate. Gender and age were included as covariates in the subsequent analysis.

### Results

#### Reaction time

The main effect of memory priming on reaction time was significant: the control group constructed EFT events faster than the EM priming group [*F*_(1, 70)_ = 1.69, *p* <0.05, η^2^ = 0.06]. Event familiarity did not significantly affect reaction time [*F*_(1, 70)_ = 0.14, *p* > 0.05, η^2^ = 0.002), and we calculated *B* using a uniform distribution, as this permitted prediction of the maximum effect (uniform from −40 to 40) rather than specifying a plausible predicted effect size P (as there are no previous studies or theory for reference). This yielded *B* = 0.01. Importantly, the interaction between familiarity and memory priming was significant [*F*_(1, 70)_ = 11.81, *p* <0.001, η^2^ = 0.014].

**Figure 1:**
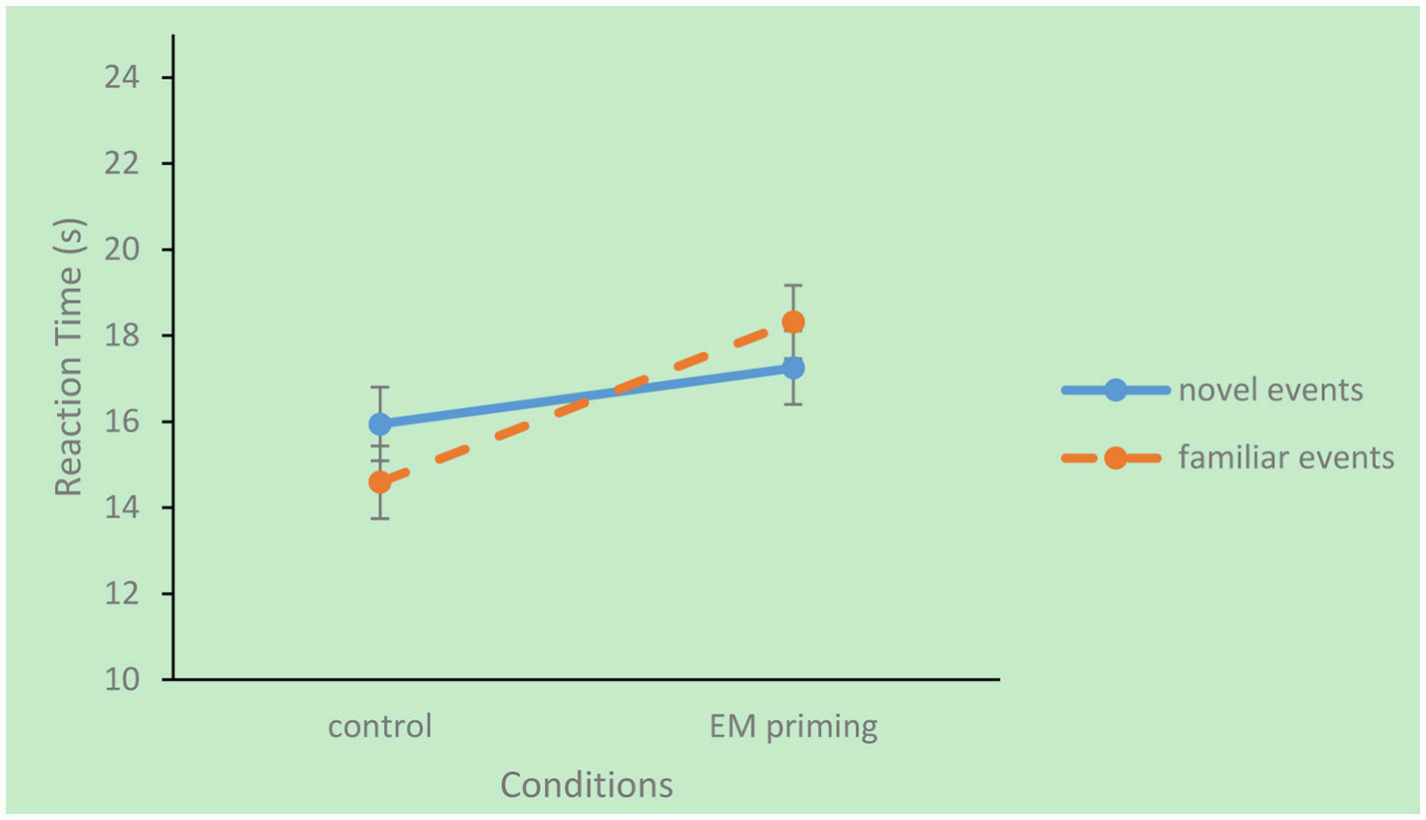
**The interaction effect of familiarity and priming condition on reaction time**.

Simple effect analysis indicated that familiar events were constructed faster than novel events in the control group (*p* = 0.009). In contrast, familiar events took longer to construct than novel events in the EM priming group (*p* = 0.03). Additionally, compared with the control group, EM priming significantly prolonged reaction time regarding familiar events (*p* = 0.003) but did not significantly affect reaction time regarding novel episodic events (*p* = 0.29; Figure 1).

#### Ratings of phenomenal characteristics

The repeated-measures ANOVA indicated that familiarity did not have a significant main effect on the number of sensorial details (Bayes' *B* = 0.05; Table 2). However, the main effect of familiarity on clarity of context and subjective experience was significant, indicating participants rated familiar EFT as clearer and involving a stronger feeling of having been experienced. The main effect of priming on all phenomenal indexes was non-significant (all values of Bayes' *B* <1/3), indicating that priming the EM system did not significantly affect the content of future events. The interaction effects between familiarity and priming on all phenomenal indexes was non-significant; however, all values of Bayes' *B* were 1/3–3, indicating insensitive data.

**Table 2:** **Phenomenal characteristics between familiarity and priming conditions**.

	**EM priming**		**Control**		**Main effects**				**Interaction**	
	**Familiar**	**Unfamiliar**	**Familiar**	**Unfamiliar**	**Priming**		**Familiarity**			
	***M(SD)***	***M(SD)***	***M(SD)***	**M(SD)**	***F***	**η^2^**	***F***	**η^2^**	***F***	**η^2^**
Sen.	3.83 (0.56)	3.98 (0.77)	3.69 (0.83)	3.75 (0.80)	1.65	0.02	2.49	0.03	0.48	0.01
Cla.	4.89 (0.77)	4.49 (0.80)	4.93 (0.79)	4.55 (0.93)	0.10	0.00	32.24^***^	0.27	0.02	0.00
Sub.	4.82 (0.84)	4.40 (0.83)	4.61 (0.85)	4.34 (1.05)	0.54	0.01	10.26^**^	0.13	0.49	0.01

*Sen., number of sensorial details; Cla., clarity of context; Sub., subjective experience*.

** *p <0.01*,

*** *p <0.001*.

The control group's ratings of EFT's phenomenal characteristics fully replicated the results of Experiment 1, which also found that familiar and novel EFTs did not differ significantly regarding the number of sensorial details [*F*_(1, 45)_ = 0.38, *p* = 0.54, η^2^ = 0.01, *B* = 0.01] or subjective experience [*F*_(1, 45)_ = 3.10, *p* = 0.09, η^2^ = 0.09, *B* = 0.29], and that familiar EFT were more clearly represented [*F*_(1, 45)_ = 14.87, *p* <0.001,η^2^ = 0.25; all statistics from Experiment 2].

In sum, in Experiment 2, EM priming mainly affected EFT regarding reaction time and preferentially affected familiar EFT. Specifically, EM priming increased reaction time for familiar but not novel EFT. The former finding supported our hypothesis; the latter interestingly did not.

## General discussion

In this study, we hypothesized that familiarity moderates EM and SM's contribution to EFT. We conducted two experiments to test this hypothesis. The results of Experiment 1 directly supported our hypothesis: both familiar and novel EFT relied more heavily on EM than SM. Experiment 2 tested the results of Experiment 1 based on the consideration that priming the EM system would interact with familiarity to affect EFT if the results of Experiment 1 were had been interpreted correctly. In Experiment 2, priming the EM system affected familiar EFT more strongly than novel EFT, further supporting our hypothesis. Collectively, these two experiments' results indicate that event familiarity moderates EM and SM's contribution to EFT.

Familiarity's moderation of EM and SM's contribution to EFT may particularly depend on whether the individual is able to draw on sufficient related episodic elements from his or her episodic memory as raw materials to construct the imagined future event. The dual-knowledge structure model proposes that episodic memories provide the episodic elements (e.g., persons, objects, locations) used to construct future events and scenarios, whereas semantic memories provide a context or frame for constructing and organizing the EFT and may also provide complementary knowledge of one's personal past (D'Argembeau, 2015). SM may be particularly important to novel EFT, as few or no prior related episodic elements may be available due to the absence of related episodic memories. In that case, the individual may depend on SM to complement his or her episodic knowledge by providing undifferentiated conceptual information that “fills in the blanks,” thereby permitting the construction of novel events (Irish and Piguet, 2013). This proposal also explains why people suffering SM impairment experience difficulty imagining novel events (Irish and Piguet, 2013). In contrast, regarding familiar EFT, sufficient related episodic details are more likely to be readily accessible, allowing the individual to use SM less. Accordingly, novel EFT showed greater reliance on SM but less on EM, compared to familiar EFT.

In Experiment 2, priming the EM system interacted with familiarity to affect EFT, further supporting the hypothesis that EM and SM differentially contribute to EFT. In the control group, familiar future events were constructed faster than the novel ones; however, priming the EM system significantly increased reaction time for familiar EFT but not novel EFT. Hence, it took much longer to construct a standard familiar future event relative to a novel one. This result is completely contrary to our hypothesis, in which we supposed that EM priming would reduce the time needed to construct familiar EFT since sufficient related episodic details were readily accessible. There are two possible explanations for this result. One is that, according to previous research, spreading activation was applied quite extensively to episodic memory research (Roediger et al., 2001; Chan et al., 2006); free recalling of a specific event may activate other related episodic memories and make these related experiences relatively accessible. It seems plausible that, when imagining familiar episodic future events, EM priming makes a greater number of episodic elements accessible, requiring individuals to spend longer selecting and extracting the most appropriate elements before recombining them into a coherent EFT. In contrast, regarding novel EFT, EM priming activates only the small number of existing related episodic elements, thus leading to no significant effect on the time to construct novel EFT. In contrast with the facilitative effect above, another possibility is that the retrieval of a past event may lead to the phenomenon of retrieval-induced forgetting, implying that free recall and elaboration inhibit the retrieval of other episodic memories (Bäuml and Samenieh, 2012), thereby preventing participants constructing future events. Since familiar EFT relies more heavily on EM, it would be affected more heavily than novel EFT. Future research should test these possibilities.

In addition, participants in Experiments 1 and 2 (regarding Experiment 2's control group) both rated familiar EFT as more clearly represented than novel EFT, although familiar and novel EFT did not differ significantly regarding the number of sensorial details or the subjective richness of experience. This result is inconsistent with some results from studies conducted outside the Chinese context which found participants have rated familiar EFT as containing more sensorial details and giving a stronger subjective experience, as well as being more clearly represented (Szpunar and McDermott, 2008; de Vito et al., 2012b). Therefore, they may partly reflect a culture difference. There are several possible explanations for this inconsistent result. First, this difference may originate in differing narrative practices pertaining to early parent-child conversation: Euro-American mothers commonly engage in highly elaborative memory conservations with their children, whereas Chinese mothers tend to engage in less elaborative memory conversations (Wang et al., 2011). We therefore suppose that the non-significant difference in sensorial detail between familiar and novel EFT may result from a Chinese tendency to attend less strongly to episodic details in general, regarding both familiar and novel events. Additionally, during interviews conducted in this study, a large number of participants reported experiencing difficultly imagining detailed future events; this may reflect a general Chinese tendency to attend less strongly to episodic details. Second, Chinese people may imagine familiar events vividly, but omit a relatively large amount of detail when asked to write it down or describe it aloud. The data used for selecting event cues in Experiment 2 and the interview after experiment partly support this possibility. Specifically, the primary data of 130 event cues showed that familiarity was significantly correlated with specificity (*r* = 0.838, *p* <0.001), implying the familiar events were imagined more vividly; additionally, during interviews some participants reported being able to imagine future events more vividly than they were able to report. Therefore, we suppose that the non-significant difference in sensorial detail between familiar and novel EFT may result from highly condensed expression in general and particularly regarding familiar EFT. Third, the visual perspective of imagination may underlie this inconsistent result. A third-person perspective was more common among participants from Eastern nations, while participants from Western cultures resonated more strongly with a first-person perspective (Christian et al., 2013). Moreover, visual perspective may influence the vividness of mental imagery. Specifically, a first-person perspective has been shown to provide greater access to the sensory experiences of a mental event, whereas a third-person perspective decreases sensorial experiences and is more likely to emphasize propositional self-beliefs (Libby et al., 2014; Christian et al., 2015, 2016). Therefore, the non-significant difference in sensorial detail between familiar and novel EFT may also reflect the fact that Chinese people more commonly use the third-person perspective, regarding familiar and novel events. Furthermore, the subjective richness of EFT is positively correlated with the vividness of episode details (Tulving, 1985; Szpunar and McDermott, 2008); accordingly, the richness of subjective experience did not differ significantly between familiar and novel EFT. Future research should test these possibilities.

This study's results indicate that familiarity moderates EM and SM's relative contribution to EFT; this extends the understanding of the relationship between memory and EFT. Nonetheless, this study has the following limitations. First, Experiment 2 did not include a SM priming condition, as the results of Experiment 1 indicated that both novel and familiar EFT depended less on SM than on EM, which may reflect EFT's “episodic” nature, and implying that SM priming marginally affects EFT. Future research should further explore this point. Second, in Experiment 2, priming the EM system may have elicited distinct emotions; these may have importantly confounded analysis of EFT construction and elaboration. Therefore, future research should separately analyze memory priming and emotion priming's effects on EFT. Third, participants were instructed to say aloud or write down everything that came to mind while they were imagining aimed to picture images in their mind; however, the influence of narration conventions effect cannot be excluded, people may spent time to structure their words in consideration of clear narration (D'Argembeau and Mathy, 2011). Additionally, we adopted the cue-word paradigm and aimed to separately examine memory priming's effect on reaction time and event's content; however, as a natural process, EFT does not necessarily proceed through construction before beginning elaboration. Future research should therefore test this paradigm's ecological validity; for instance, by using event-related potentials and fMRI to examine covariance in EM- and SM-related regions during EFT and test if familiarity moderates the activation of this functional network.

## Author contributions

TW designed and conducted the experiment protocol, analyzed the data, and drafted this manuscript; TY participated in the development of the encoding principle and reviewed the manuscript; XH reviewed the manuscript and provided important comments and revision. All authors approved the final manuscript.

## Funding

This research was supported by the National Natural Science Foundation of China (31600879) and the Humanities and Social Science Research Project of Chongqing (14SKB008).

### Conflict of interest statement

The authors declare that the research was conducted in the absence of any commercial or financial relationships that could be construed as a potential conflict of interest. The reviewer XG declared a past collaboration and co-authorship with one of the authors XH to the handling Editor, who ensured that the process met the standards of a fair and objective review.

## Footnotes

^1^We did not collect familiarity scores after completing the EFT task (as in Experiment 1) as EM priming spanning many intervening items would impair its priming effect, and Experiment 1 have showed the results of pre-test and post-test were consistent.
